# Training and Recruitment to Implement the CASA Psychosocial Intervention in Cancer Care

**DOI:** 10.3390/ijerph23010116

**Published:** 2026-01-17

**Authors:** Normarie Torres-Blasco, Stephanie D. Torres-Marrero, Ninoshka Rivera-Torres, Denise Cortés-Cortés, Sabrina Pérez-De Santiago

**Affiliations:** 1 School of Behavioral and Brain Sciences, Ponce Health Sciences University, Ponce 00716, Puerto Rico; storres21@stu.psm.edu (S.D.T.-M.); nirivera23@stu.psm.edu (N.R.-T.); dcortes24@stu.psm.edu (D.C.-C.); sperez24@stu.psm.edu (S.P.-D.S.); 2 Ponce Medical School Foundation Inc., Ponce Research Institute, Ponce 00716, Puerto Rico

**Keywords:** Meaning-Centered Psychotherapy, doctoral students, community partners, advanced cancer, dyads, Consolidated Framework for Implementation Research, training

## Abstract

**Highlights:**

**Public health relevance—How does this work relate to a public health issue?**

- Latino patients with advanced cancer and their caregivers face high psychosocial and spiritual distress, compounded by barriers to accessing culturally responsive psycho-oncology care.
- This study addresses the public health need for scalable training and recruitment strategies to support equitable implementation of psychosocial interventions in cancer care settings.

**Public health significance—Why is this work of significance to public health?**

- Findings demonstrate that training doctoral students and community partners supports early implementation of a culturally adapted psychosocial intervention for Latino cancer dyads.
- Integrating institutional infrastructure with community-based recruitment highlights complementary pathways to improve reach and engagement in underserved populations.

**Public health implications—What are the key implications or messages for practitioners, policy makers and/or researchers in public health?**

- Early investment in structured training and community partnerships may strengthen sustainable delivery of psycho-oncology interventions in resource-limited settings.
- Implementation-focused pre-pilot studies can inform scalable models for integrating psychosocial care into oncology services while advancing health equity.

**Abstract:**

Practical training and recruitment strategies are critical for the sustainable implementation of psychosocial interventions. However, few studies have examined how to prepare community partners and doctoral students to support culturally adapted psycho-oncology interventions. This pre-pilot study aims first to evaluate two distinct training programs and recruitment procedures, and second to explore preliminary pre-post outcomes of the Caregiver-Patients Support to Cope with Advanced Cancer (CASA) intervention, using the Consolidated Framework for Implementation Research (CFIR). Three clinical psychology graduate students received CASA training, and two community partners completed Recruitment training. We present descriptive pre- and post-assessments, along with qualitative feedback, for both training and institutional (Puerto Rico Biobank) and community-based recruitment outcomes. A related-samples nonparametric analysis examined pre- and post-CASA intervention signals. Results indicated knowledge gains among doctoral students (pre-test M = 3.3; post-test M = 9.3) and community partners (pre-test M = 4.5; post-test M = 9.5). Preliminary outcomes revealed significant improvements in spiritual well-being (*Z* = −2.618, *p* = 0.009) and quality of life (*Z* = −2.957, *p* = 0.003) and a reduction in depressive (*Z* = −2.764, *p* = 0.006), anxiety (*Z* = −2.667, *p* = 0.008), and distress (*Z* = −2.195, *p* = 0.028) symptoms following CASA. Of 26 recruited dyads, institutional referrals enrolled 16 dyads (61.5%), while community partners referred 10 dyads with a 90.9% success rate. Findings support the feasibility of both training and CASA exploratory outcomes suggest meaningful psychosocial benefits for Latino dyads coping with advanced cancer. Combining institutional infrastructure with community engagement may enhance sustainability and equitable access to psycho-oncology care.

## 1. Introduction

Patients with advanced cancer often experience profound emotional, psychological, and existential distress [1,2,3]. Alongside physical symptoms, individuals frequently face depression, anxiety, fear of death, loss of meaning, and disrupted communication with loved ones. These psychosocial challenges can significantly diminish quality of life, impact treatment adherence, and exacerbate feelings of isolation [1,2,3,4,5]. As a result, addressing the emotional and spiritual needs of patients with advanced cancer has become a critical component of comprehensive cancer care. Spirituality is a multifaceted construct that encompasses meaning, peace, and faith, and does not necessarily depend on religious affiliation [6]. The concept of spirituality can involve a sense of connection with God, nature, other individuals, and the environment, which is associated with quality and meaning in life [7]. Implementing meaning and peace approaches to address spiritual needs in cancer patients and alleviate their existential distress has become increasingly important [8,9]. To address this need, William Breitbart and colleagues developed Meaning-Centered Psychotherapy (MCP) as a psychotherapeutic intervention to enhance a sense of meaning, peace, and purpose in patients with advanced cancer [10,11].

Breitbart developed Meaning-Centered Psychotherapy based on Viktor Frankl’s principles of existential psychology and philosophy, emphasizing that meaning is a primary source of human motivation and can help people cope with adverse situations [12,13]. MCP differs from other types of psychotherapy in that it aims to identify sources of meaning in the patient’s life through a set of strategies that focus on the meaning of life, the will to find meaning, freedom of will, and sources of meaning [6]. Interventions such as MCP have showcased positive results on patients’ spiritual needs by enhancing their sense of meaning, peace, and purpose while confronting terminal illness [6]. Likewise, a systematic review found that MCP is efficacious in improving spiritual well-being, quality of life, sense of meaning, and psychological distress [14].

The MCP was adapted to include family caregivers, thereby becoming a dyadic intervention. The adapted MCP, named Caregiver-Patients Support to Cope with Advanced Cancer (CASA), is a culturally tailored psychosocial intervention designed to enhance communication between patients and their caregivers and to foster their spiritual well-being [15]. CASA integrates components of the MCP and the Communication Couple Skill Training (CCST) and comprises five manualized sessions [16]. During its cultural adaptation, both patients and caregivers reported that CASA was acceptable and culturally relevant [15]. A prior study also underscored the importance of involving community partners in the referral process and establishing long-term sustainability strategies early in their implementation [15,17]. Consistent with health equity and implementation literature, community engagement and external partnerships are recognized as foundational to sustainable, community-based interventions [18,19,20].

Recruitment strategies and workforce training are critical components for the successful implementation and dissemination of psychosocial interventions [21]. Training doctoral students equips future clinicians to deliver evidence-based interventions that address the emotional and spiritual needs of patients and caregivers [10]. At the same time, community partners play a vital role in facilitating access and engagement in underserved populations [17,20]. However, despite their recognized importance, limited work has examined how the training of clinical psychology doctoral students and community partners, together with recruitment strategies, functions as an integrated component of early implementation of culturally adapted psychosocial interventions such as CASA. As a result, there remains a gap in understanding how the early implementation process shapes the potential to preserve effectiveness and ensure successful long-term implementation. Addressing this gap requires implementation-focused evaluation, guided by frameworks such as the Consolidated Framework for Implementation Research (CFIR), which can inform refinement, scalability, and sustainability of culturally adapted interventions.

### Consolidated Framework for Implementation Research (CFIR)

The CFIR provides an overarching typology to support the development of implementation theories and examine what works where and why in different contexts [22,23]. This framework encompasses five main domains that interact dynamically to influence implementation effectiveness: intervention characteristics, individual characteristics, inner and outer settings, and process [23]. In this study, CFIR is used to frame the examination of early implementation factors relevant to the CASA intervention, specifically at the construct level applicable to each domain. The intervention characteristics domain focuses on the features and quality of the intervention, as well as on the perceptions of those responsible for behavioral changes [23]. Within the intervention characteristics domain, this study focuses on adaptability and design quality, which are central to understanding how training content can be adjusted to support early uptake across settings and modalities. The characteristics of the individual domain refer to personal factors of those engaged in the implementation, which guide the examination of knowledge, beliefs, and self-efficacy of participants involved in the training. In this study, both domains informed the evaluation of two distinct training programs for community partners and clinical psychology doctoral students, with emphasis on feasibility and acceptability, knowledge gains, and perceived preparedness. Moreover, the intervention and individual characteristics domain also encompasses preliminary outcomes of the CASA intervention for patients with advanced cancer and their caregivers.

The inner setting domain encompasses the organizational and contextual factors that shape implementation, including network and communication, leadership engagement, and available resources. In our context, this included engaging the Puerto Bio-Bank (PRBB) and community partners as a recruitment source. The outer setting domain encompasses external influences, and in this study, we emphasize alignments with patients’ needs and resources [15]. Finally, the process domain centers on the planning and evaluation of implementation activities by examining both training and recruitment procedures.

Guided by the CFIR framework, this study aims to evaluate critical factors during the early implementation of the CASA intervention. Specifically, the objectives are to: (1) assess the knowledge improvement with two distinct training programs—one on recruitment strategies for community partners and one on CASA training for doctoral students, and to examine the recruitment outcomes from two sources —PRBB and community engagement, and (2) explore the preliminary pre-post change in emotional and spiritual well-being among CASA participants. Accordingly, this study is framed as a formative implementation pre-pilot, with intervention outcomes examined as exploratory signals rather than confirmatory evidence. By situating these objectives within the CFIR domains, the study seeks to support the sustainable implementation of the CASA intervention.

## 2. Materials and Methods

**Figure 1:**
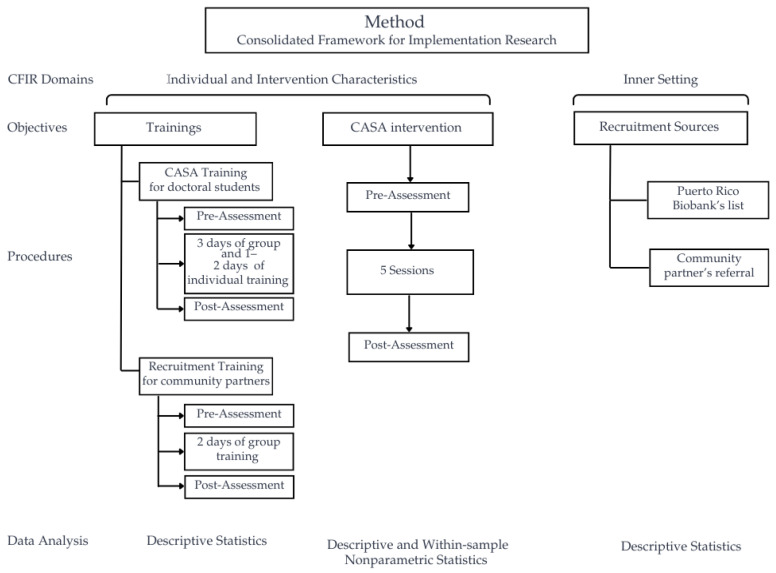
Flowchart of study methods and procedures.

This article presents a formative implementation pre-pilot study guided by the Consolidated Framework for Implementation Research (CFIR). The primary focus of the study is to examine training and recruitment procedures during the early implementation of the CASA intervention by evaluating knowledge improvement associated with the two training programs (CASA training and recruitment training) and to describe the recruitment outcomes from institutional and community sources. As a secondary exploratory component, the study also examined preliminary outcomes for patients and caregivers who received the CASA intervention. Figure 1 provides an overview of the study design, including training, CASA intervention, assessments, recruitment sources, and analysis.

### 2.1. Study Design

This formative pre-pilot implementation study aimed to evaluate changes in participants’ knowledge of both the CASA intervention and Recruitment training and to analyze the success of two recruitment sources. Additionally, we explore the preliminary pre-post changes in emotional and spiritual well-being of the CASA intervention. These results will inform its early implementation on a broader trial. We used a descriptive pre- and post-test design to measure knowledge gains and to collect qualitative feedback from participants regarding the training. Similarly, the assessment of recruitment success employed a descriptive approach, focusing on the proportion of participants recruited for the CASA intervention. Furthermore, we conducted a comparison within-sample using a pre-/post design to explore the preliminary outcomes of the CASA intervention. All study procedures were reviewed and approved by the Institutional Review Board (IRB) of Ponce Health Sciences University/Ponce Research Institute (IRB protocol #2310171716). All participants provided informed consent before participating.

Although the study design was not structured around the Consolidated Framework for Implementation Research (CFIR), the framework informed the selection and organization of data during the early phase of implementation. As a formative pre-pilot implementation study, we used the CFIR to structure the presentation and interpretation of findings across domains and constructs relevant to training, recruitment, and early intervention delivery.

### 2.2. Participants

Participants who completed the training included two community partners and three doctoral students in clinical psychology. The community partners who participated in the recruitment training were from Puerto Rico’s southern region and were actively engaged in their communities. The doctoral students who completed the CASA training were currently enrolled in the Clinical Psychology program at Ponce Health Sciences University and had at least two years of clinical practice experience. Furthermore, the inclusion criteria for the CASA intervention included patients diagnosed with stage III or IV cancer, a Distress Thermometer (National Comprehensive Cancer Network, Plymouth Meeting, PA, USA) score of 4 or higher, and a caregiver willing to participate. We used the Distress Thermometer, as it is commonly used in oncology and mental health settings to identify patients who may benefit from a more in-depth psychological evaluation [24]. Both patients and caregivers must be 21 years of age or older. The exclusion criteria to participate in the intervention were individuals diagnosed with a major disabling medical condition, unable to understand the consent procedures, or too ill to participate. All participants were recruited through convenience and availability sampling and provided their consent to participate in the training as part of the study. Each community partner, doctoral student, and dyad (patient-caregiver) received $50 for completing each assessment, as compensation for their time and effort in participating in the study.

### 2.3. Procedures

In this study, we delivered two distinct training sessions: one for doctoral students and the other for community partners. The doctoral student training focused on the CASA intervention, equipping them with the knowledge and skills needed to deliver it. In contrast, the training for community partners centered on recruitment strategies to enhance community engagement and referral processes. Additionally, we implemented the CASA intervention in a small sample to explore its preliminary outcomes.

#### 2.3.1. CASA Training

The doctoral students received structured training in both group and individual modalities, equipping them with the knowledge and skills to deliver the CASA intervention. Table 1 summarizes the CASA training structure. The principal investigator of this study (N.T.-B.), a licensed clinical psychologist in Puerto Rico, provided CASA training to the clinical psychology doctoral student.

The group training lasted three weeks, one day per week. Day 1 began with an overview of the foundational components of the CASA intervention, which integrates Communication Couple Skill Training (CCST) and an adaptation of individual Meaning-Centered Psychotherapy (MCP) for Latino/Hispanic populations. The supervisor introduced the structure and content of each MCP and CCST session. On this day, after explaining sessions with similar content included in the CASA intervention, students were expected to complete at least one session of the MCP or CCST. During these role-plays, the supervisor acted as the patient and the student as the interventionist, using the same case across all sessions. Trainees were assigned to study the CASA manual in preparation for Day 2 of training. Day one lasted approximately six hours.

Day 2 (four hours) focused solely on the CASA intervention. The supervisor provided detailed instructions on session delivery, clarified questions about the protocol, and discussed the fidelity checklist, which outlined the specific tasks expected of trainees for each session. The supervisor led standardized role-plays based on a case she developed, in which N.T.-B. portrayed the patient; a non-trainee individual served as the caregiver; and the trainees served as interventionists. One student performed a role-play of CASA Session 1. Students continued reviewing the manual independently between training sessions.

Day 3 focused on conducting role-plays for CASA Sessions 2, 3, and 4, with at least one student performing a complete session for each. This day lasted three hours, with about one hour per session. The role-plays followed the same case scenario used in CASA Session 1, allowing for continuity and cumulative skill development. For each role-play during the group training, one trainee performed while others observed, fostering peer learning, reflection, and collaborative feedback. The supervisor provided immediate supervision and feedback after each exercise, and students were encouraged to ask questions about the role-plays they performed or observed.

In addition to group training, each student completed an individual role-play session on a separate day. During this session, each trainee conducted CASA Session 5 under direct supervision, receiving individualized feedback to reinforce adherence to the CASA protocol. The supervisor or the trainee could request an additional individual role-play and supervision session if needed. Multiple resources supported the training, including an independent review of the CASA manual, group discussions, individual supervision, and video recordings of training sessions, which allowed trainees to revisit the material as needed.

#### 2.3.2. Recruitment Training

The principal investigator (N.T.-B.) delivered the recruitment training in two sessions, each lasting 2 to 3 h. See Table 2 for a summary of the recruitment training content and activities. On Day 1, community partners participated in a didactic and interactive session that covered the ethical principles of human subjects’ research, including federal definitions of research, human subjects, intervention, and interaction as outlined in 45 CFR 46 [25]. The training emphasized the role of Institutional Review Boards (IRB), confidentiality, and privacy expectations when collecting or handling identifiable information. In addition, the training also incorporated the purpose of the CASA intervention study, inclusion criteria, and the use of the Distress Thermometer to assess emotional distress in patients coping with advanced cancer. On this first day, the session also introduced Motivational Interviewing (MI) techniques, emphasizing open-ended questions, reflective listening, and affirmations to explore ambivalence and enhance patients’ motivation to participate in the study. MI techniques were used because evidence supports their effectiveness in improving participants’ retention and attendance in the intervention [26].

On Day 2, community partners practiced these MI strategies through supervised role-play exercises that also included administering the Distress Thermometer. Role-play scenarios simulated common recruitment challenges, such as engaging a willing patient, addressing denial, and navigating ambivalence. The research team developed three distinct cases: a patient interested in participating, a patient ambivalent about participating, and a patient in denial of participating. Each role-play involved one community partner acting as the recruiter and a member of the research team simulating the patient. The PI provided immediate feedback after each exercise, highlighting the importance of empathy, validation, and maintaining non-pressured communication during recruitment conversations. These exercises allowed community partners to apply newly acquired skills and refine their communication skills in a supervised, interactive setting, and to strengthen their confidence in recruiting participants for the intervention.

#### 2.3.3. CASA Intervention

The CASA intervention is a dyadic intervention for both patients coping with advanced cancer and their caregivers. The goal of the intervention is to enhance spiritual well-being and communication within the dyad. The CASA intervention includes five manualized sessions: (1) an introductory session to explore treatment goals and the cancer experience, (2) “Identity and Communication Skills,” (3) “Creative Sources of Meaning,” (4) “Experiential Sources of Meaning,” and (5) a closing transition session focused on integration and commitment to life. Table 3 presents an overview of the CASA intervention. Each session combines psychoeducation, experiential exercises, and reflective dialog to foster a shared understanding, mutual support, and meaning-making within the dyad [11]. In this study, the clinical psychology doctoral students who received the CASA training delivered the CASA intervention via telemedicine (telephone sessions). Each session lasts 45 to 60 min over 5 to 10 weeks, once weekly or biweekly, depending on patients’ and caregivers’ availability.

### 2.4. Measures

#### 2.4.1. Training Assessments

Before and after each training, participants completed an assessment that measured their change in knowledge regarding the content of each distinct training. These assessments were training-specific and designed to evaluate feasibility and participant comprehension of core training content, rather than as validated outcome measures. The CASA Training pre- and post-assessment consisted of 10 true-or-false questions, developed by the research team, and aligned with the CASA training learning objectives, to evaluate participants’ knowledge of the CASA intervention content, core components, and delivery structure. Item development was informed by intervention expert input from the principal investigator. Some examples of the assessment items included: (1) “The CASA intervention can be delivered individually,” and (6) “The ‘commitment to life’ strategy helps patients and caregivers find meaning regardless of their circumstances”. Both pre- and post-assessments were completed in paper-and-pencil format immediately before (Day 1) and after the last individual training session for each student.

Similarly, the Recruitment Training pre- and post-assessment consisted of 10 true-or-false questions, developed by the research team, and aligned with the training learning objectives, to evaluate participants’ knowledge of human subjects and research procedures. These assessments included items such as (5) “A ‘human subject’ in a study is someone from whom information or samples are obtained”, and (10) “A physician or nurse who treats patients can be a useful source of information for cancer-related studies”. Community partners completed both pre- and post-assessments in paper-and-pencil format on Day 1 (before training) and on Day 2 (after training).

Each assessment had a maximum score of 10 points, with one point awarded for each correct answer. The research team instructed participants to respond to each statement by indicating whether they believed it was true or false, according to their understanding of the content. To ensure that participants met the training competency requirement, we established a minimum benchmark of scoring at least 80% correct on the post-assessment. The assessment was not formally psychometrically validated.

#### 2.4.2. CASA Intervention Measures

To assess the CASA intervention outcomes, both patients and caregivers independently completed pre- and post-assessments. Participants completed these assessments by phone at least 1 week before starting the intervention and after 1 or 2 weeks of completing the intervention. The CASA intervention measures encompassed several questionnaires assessing spiritual well-being, emotional symptoms, and quality of life. We used the Functional Assessment of Chronic Illness Therapy—Spiritual Well-Being Scale 12 (FACIT-sp-12; FACIT.org, Ponte Vedra, FL, USA) to assess individual spiritual well-being. This measure is a brief self-report, divided into two subscales: spirituality and meaning/peace [6]. To measure depressive symptoms, we used the Patient Health Questionnaire-8 (PHQ-8; Pfizer Inc., NY, USA). The PHQ-8 is a self-report instrument of 8 items that assesses the frequency of depressive symptoms over the past 2 weeks. In Puerto Rico, this scale has demonstrated high internal consistency (α = 0.92) [27]. We assessed anxiety symptoms through the Generalized Anxiety Disorder 7 (GAD-7; Pfizer Inc., NY, USA). The GAD-7 has demonstrated excellent psychometric properties, including high internal consistency (α ≈ 0.91) in Puerto Rican populations [28]. Furthermore, we included the Distress Thermometer, which quickly assesses a person’s overall level of emotional distress [29]. Finally, we used the Functional Assessment of Cancer Therapy-General (FACT-G, FACIT.org, Ponte Vedra, FL, USA) to evaluate health-related quality of life. The FACT-G is composed of 28 items designed to rate physical, social, family, emotional, and functional well-being, in which a higher total score represents a better general quality of life [30].

### 2.5. Recruitment Sources for the Pre-Pilot Study

The research team recruited participants for the pilot study through two complementary sources: institutional recruitment via the Puerto Rico Biobank (PRBB) and community-based recruitment through trained community partners. The team implemented both recruitment sources to ensure diverse participant representation and to evaluate recruitment success among both approaches.

The Puerto Rico Biobank (PRBB) served as our only institutional recruitment source. Established in 2009, the PRBB is a cancer-focused biobank that collects biospecimens and associated data from patients in southern Puerto Rico to address cancer-related health disparities [31]. For this study, the PRBB provided a list of patients coping with advanced cancer, which is one of our patient eligibility criteria. The research team then initiated initial contact with these patients by phone, explained the study, and screened them according to the CASA inclusion criteria. This structured process ensured standardized and ethical recruitment within the institutional setting.

The community-based recruitment relied on the active engagement of community partners, who applied the strategies learned in the recruitment training, such as motivational interviewing and administering the Distress Thermometer, to identify and refer eligible participants. These tools allowed partners to assess patients’ emotional distress and determine potential patients’ eligibility. Once the community partner confirmed the participants’ interest and eligibility, they shared the participants’ contact information with the study team, which then reached out to verify their enrollment and address any questions or concerns.

To address recruitment challenges and strengthen community buy-in, the principal investigator (N.T.-B.) wrote letters of collaboration for non-profit organizations, such as the American Cancer Society. These letters informed organizations about the study, promoted institutional support, and encouraged referrals, thereby fostering trust and expanding the reach of recruitment efforts within the broader community.

### 2.6. Data Analysis

The training and recruitment data presented in this study were analyzed using descriptive statistics to summarize changes in knowledge and recruitment outcomes. For both training programs, we used a pre- and post-test descriptive design to report mean scores, calculated manually, to represent changes in knowledge. Additionally, both community partners and doctoral students provided feedback on their training via a survey. The research team presented participants’ feedback descriptively to capture general impressions and highlight common observations. No formal qualitative coding or software was used, given the limited amount and exploratory nature of the feedback collected.

The recruitment outcomes were summarized descriptively by manually calculating the number and percentage of participants enrolled through each recruitment source: the Puerto Rico Biobank and community leaders. These data provided insight into the feasibility of institutional and community-based recruitment strategies during the early implementation of the CASA intervention. All analyses aimed to inform future refinement of the CASA implementation protocol and to optimize recruitment and training strategies for subsequent pilot testing.

We conducted a two-related sample comparison to examine preliminary pre–post changes in patient and caregiver outcomes following the CASA intervention. Because of the small sample size and the non-normal distribution of the outcome variables, we used the Wilcoxon Signed-Rank Test, a nonparametric paired-samples procedure appropriate for evaluating within-group change [32]. The research team computed pre-post comparisons for spiritual well-being (FACIT-Sp-12), depressive symptoms (PHQ-8), anxiety symptoms (GAD-7), quality of life (FACT-G), and emotional distress (Distress Thermometer), using SPSS v.31. For each measure, we reported means, standard deviation, medians, and Wilcoxon test statistics (Z values) with corresponding *p*-values and effect size of those cases that completed both pre- and post-assessment. Given the small sample size and the number of outcomes examined, analyses were conducted to inform future research within the context of a formative, pre-pilot study. We applied the Holm–Bonferroni procedure to adjust significance thresholds across outcomes, and findings should be interpreted in the context of early implementation. We also performed descriptive statistics to present sociodemographic characteristics.

### 2.7. Consolidated Framework for Implementation Research Operationalization

The Consolidated Framework for Implementation Research (CFIR) was used as an analytic framework to guide the operationalization and interpretation of this formative pre-pilot implementation study, encompassing training, recruitment, and early intervention outcomes. Specific CFIR domains and constructs were prospectively linked to data sources, measures, and analytic approaches.

The intervention characteristics domain was operationalized through the constructs of adaptability and design quality and packaging. These constructs were informed by trainees’ qualitative feedback on the clarity, structure, and perceived usefulness of the CASA and recruitment training, as well as the intervention’s flexibility to accommodate delivery needs during early implementation. The characteristic of an individual’s domain was operationalized through the constructs of knowledge and beliefs about the intervention and self-efficacy related to both training and intervention delivery. Knowledge gains were assessed using pre- and post-training assessments completed by doctoral students (CASA training) and community partners (recruitment training). Self-efficacy was examined through trainees’ qualitative feedback, which reflected confidence, perceived preparedness, and identified areas for further skill development. In addition, preliminary pre–post changes in patients’ and caregivers’ psychosocial outcomes following the CASA intervention were examined as exploratory indicators to inform perceptions of the intervention’s relevance and potential benefits during early implementation.

The inner setting domain was operationalized by examining networks and communication, leadership engagement, and available resources. Networks, communication, and leadership engagement were assessed by examining the structure and reach of recruitment channels, including reliance on the Puerto Rico Biobank (PRBB) and trained community partners, as well as the relative contributions of institutional versus community sources. Available resources were operationalized as the number of trained interventionists and active community partners available to support intervention delivery and recruitment during this early phase. The outer setting domain was operationalized through the construct of patients’ needs and resources by aligning CASA intervention content and eligibility criteria with participants’ psychosocial distress, as reflected in the inclusion of a Distress Thermometer score ≥ 4. Finally, the process domain was operationalized through the constructs of planning and champions. Planning was reflected in the development and delivery of structured training protocols and recruitment procedures described in the Methods section. The PRBB functioned as an institutional champion for recruitment, enabling examination of the benefits and limitations of relying on a single recruitment champion during early implementation.

Consistent with this operationalization, the Results section presents findings on the intervention characteristics and the characteristics of individual domains, based on pre- and post-training assessments and preliminary CASA intervention outcomes. Findings on inner setting are presented through recruitment rates from institutional and community-based sources, while the remaining CFIR domains are further contextualized in the Discussion section.

## 3. Results

Results are presented according to the three domains of the Consolidated Framework for Implementation Research (CFIR): intervention characteristics, individual characteristics, and inner setting. This structure enables a systematic presentation of findings from both the CASA and recruitment training, recruitment outcomes, and preliminary outcomes of the CASA intervention. The intervention and individual characteristics domains describe participants’ knowledge gains and feedback following the training sessions, as well as the CASA intervention and preliminary outcomes. The inner setting domain encompasses contextual and operational elements, including institutional support, community engagement, and recruitment outcomes. Additionally, we present the sociodemographic characteristics of trainees and the CASA intervention participants.

### 3.1. Sociodemographic Characteristics

Regarding sociodemographic characteristics, this study included participants who completed the training, as well as patients and caregivers who received the CASA intervention. Within the two distinct training—CASA training and Recruitment training—participants were three clinical psychology doctoral students and two community partners. All participants were female. Moreover, in this study, we recruited 52 participants, but only 11 dyads received the CASA intervention, comprising 11 patients and 11 caregivers. The majority of CASA participants were female, married, and had a mean age of 58 years. Additionally, most reported completing a high school education and having government and private medical insurance. Also, reported annual incomes ranged from $23,367 to $25,963; while patients indicated this amount was sufficient, caregivers reported it was not. Table 4 presents sociodemographic information for the entire recruited sample and for participants who received the CASA intervention.

### 3.2. Individual and Intervention Characteristics Domains

Trainee participants completed pre- and post-assessments before and after the two distinct trainings—the CASA intervention and the Recruitment training—to descriptively examine changes in their knowledge of each program’s focus. These are presented within the Intervention Characteristics (design quality and packaging construct) and the Individual Characteristics domain (knowledge, belief, and self-efficacy). In the CASA intervention training, the knowledge and belief constructs were reflected in an increase in clinical psychology doctoral students’ knowledge of the CASA intervention content. The pre-test mean score was 3.3 out of 10, whereas the post-test mean score was 9.3. These descriptive results suggest that the structured training and supervised role-play sessions enhanced student mastery of the intervention content. Moreover, qualitative feedback informed the design quality and packaging construct. Doctoral students indicated that they valued the supervisor’s demonstration prior to each role-play. Additionally, students expressed interest in participating in additional role-play opportunities across sessions, noting that extended practice could further strengthen skill acquisition, reflecting aspects of self-efficacy. Box 1 presents the feedback from doctoral students.

Box 1 **Doctoral students’ qualitative feedback.**

**Verbatims**

“I consider that the training was effective as it was provided.”

“Roleplays, mainly session 3: creative sources of meanings.”

“Demonstrations of each session prior to roleplays.”

“The MCP expert demonstrates how it is done and then does the roleplays.”

“That all interventionists can carry out more sessions.”

Within the knowledge-and-belief construct of the recruitment training, community partners demonstrated increased knowledge of recruitment strategies. The mean pre-test score was 4.5 out of 10, and their mean post-test score was 9.5. Qualitative feedback, which informed the self-efficacy and design quality and packaging constructs, indicated that some partners found the training both helpful and interesting. However, they also reported that engaging with patients can be challenging. We presented representative verbatim comments in Box 2.

Box 2 **Community partners’ qualitative feedback.**

**Verbatims**

“That patients whose condition is not so advanced be included”

“Interacting with patients can be a bit difficult and frustrating at times.”

“I think it helped me.”

“I find it interesting.”

Preliminary pre–post analyses using the Wilcoxon Signed-Rank Test examined exploratory indicators of individuals’ responses to the CASA intervention during early implementation. Preliminary analysis demonstrated statistically significant improvements in several patient and caregiver outcomes (*n* = 18) following the CASA intervention. Spiritual well-being (FACIT-Sp-12) increased from a median score of 40.00 to 42.00 (*Z* = −2.618, *p* = 0.009, adjusted α = 0.025, *r* = 0.44), indicating enhanced meaning, peace, and overall spiritual well-being. Also, median quality-of-life scores (FACT-G) improved from 70.50 to 81.00 (*Z* = −2.957, *p* = 0.003, adjusted α = 0.01, *r* = 0.51), corresponding to a large effect size and suggesting broader psychosocial benefits for both patients and caregivers.

Additionally, results reflected significant reductions in emotional symptoms. Depressive symptoms (PHQ-8) decreased, with a median score rating of 7.5 to 2.00 (*Z* = −2.764, *p* = 0.006, adjusted α = 0.0125, *r* = 0.46). Anxiety symptoms (GAD-7) showed similar reductions, with a median rating from 6.00 to 1.50 (*Z* = −2.667, *p* = 0.008, adjusted α = 0.0166, *r* = 0.44). Across both instruments, baseline median scores were in the mild range, while post-intervention median scores were in the minimal range, indicating reductions that crossed established clinical severity thresholds. Similarly, the median emotional distress score measured by the Distress Thermometer declined from 5.50 to 3.00 (*Z* = −2.195, *p* = 0.028, adjusted α = 0.05, *r* = 0.38). These differences in depression, anxiety, and emotional distress reflected a medium effect size. Together, these statistically significant improvements suggest that, preliminarily, the CASA intervention may contribute to greater spiritual well-being and quality of life, while also reducing anxiety, depression, and emotional distress among Latino patients with advanced cancer and their caregivers. See Table 5 for the descriptive statistics and comparisons of pre- and post-assessments regarding preliminary signals of the CASA intervention.

### 3.3. Inner Setting Domain

Within the inner setting domain, recruitment outcomes reflect organizational factors related to network and communication and leadership engagement, which shaped the early implementation of CASA. Institutional recruitment was supported through the collaboration of Puerto Rico Bio-Bank (PRBB). Of the 26 dyads (patients and caregivers) enrolled in the pilot study, we recruited 16 dyads (61.54%) through the PRBB. From a list of 94 patients coping with advanced cancer provided by the PRBB, the research team recruited only 17.02% of them. Nearly half of the listed patients (46.81%) did not meet the inclusion criteria or declined participation, while 36.17% did not respond to contact attempts. These findings highlight the importance of institutional infrastructure in facilitating recruitment (network and communication construct), while also underscoring challenges in engaging a broader eligible population. In Table 6, we presented the frequencies and percentages of the Bio-Bank recruitment site.

Community partners contributed to participant referrals by applying the recruitment strategies introduced during training. Of the 26 dyads enrolled in the pilot study, community partners referred 10 (38.46%) patients. In total, community partners made 11 referrals, of which 10 (90.91%) resulted in successful recruitment and 1 (9.09%) did not respond. These findings show that although community-based recruitment yielded fewer referrals than institutional sources, the success rate of recruited referrals was notably high. Additionally, highlight the potential of leadership engagement to facilitate recruitment, provided that sustained engagement and structural support are in place. Table 7 presents frequencies and percentages regarding community partner referrals. Finally, when evaluating recruitment outcomes across referral sources, we found that 16 dyads (61.54%) were recruited through the Puerto Rico Biobank and 10 dyads (38.46%) through community partners. These findings underscore the need to integrate both institutional structure and community engagement into the recruitment procedures of the CASA intervention.

## 4. Discussion

This formative pre-pilot study examined the early implementation process of the CASA intervention, with a primary focus on training and recruitment. We also explored preliminary outcomes of the CASA intervention as exploratory indicators. Using the CFIR framework and its constructs enabled us to evaluate training outcomes and the recruitment process, and secondarily to examine therapeutic signals of the CASA intervention. Targeted training for doctoral students and community partners results in knowledge gains. Also, preliminary results of the CASA intervention suggest potential improvement in spiritual well-being, emotional symptoms, and quality of life for patients coping with advanced cancer and their caregivers.

Within the intervention characteristics domain, findings related to design quality and the packaging construct suggest that both the CASA intervention and recruitment training were beneficial for doctoral students and community partners. Community partners valued the cultural relevance of the training, but they also noted difficulties when approaching patients who were hesitant or in denial. Their perspective reinforces the importance of considering both professional and lived experiences when tailoring training, thereby ensuring alignment with the adaptability construct [33,34]. Preliminary findings, although based on a small sample, suggest initial signals that CASA may improve well-being and overall quality of life for both patients and caregivers, while reducing symptoms of anxiety, depression, and emotional distress. These results are consistent with prior psychospiritual interventions in advanced cancer, which underscore the importance of emotional and mental health support during the advanced cancer coping process [35,36,37]. Within CFIR, these therapeutic outcomes align with the individual’s characteristics domains, as they reflect participants’ responses to the core components of CASA. Moreover, related to the patient’s needs and resources construct in the outer setting domain, preliminary outcomes of the CASA intervention align with the intervention’s focus on addressing emotional and spiritual distress among Latino patients with advanced cancer and their caregivers.

Within the domain of individual characteristics, our findings pertained to the knowledge, belief, and self-efficacy constructs. Doctoral students who completed CASA training demonstrated increased knowledge from pre-test to post-test. Moreover, participants’ experience may have shaped their engagement with the training. Doctoral students who received the training should have at least two years of clinical experience. Consistently, the literature suggests that professional experience supports the uptake of new interventions [38]. Similarly, community partners improved their knowledge of recruitment strategies. These findings support the potential of targeted training in enhancing specific knowledge areas [39]. With respect to the self-efficacy construct, role-plays and modeled sessions were particularly helpful in supporting their learning, consistent with previous work highlighting experiential strategies as effective for psychosocial training [40]. In both training, the inclusion of roleplays, particularly those based on real patient cases, is crucial for providing practical, hands-on experience that strengthens the application of learned skills [41]. By simulating real-world scenarios, participants can practice responding to diverse patient situations, enhancing their ability to communicate effectively and address individual needs [41]. Doctoral students as interventionists support early implementation and offer a viable approach for integrating CASA into clinical training settings. This supervised trainee-delivery model is consistent with the literature, which demonstrates that well-trained trainees can effectively implement structured, manualized interventions when provided with appropriate oversight [38].

Findings related to the inner setting domain underscore the roles of networks, communication, and leadership engagement in shaping recruitment outcomes. Our findings demonstrate that institutional support, specifically the Puerto Rico Biobank, played a central role in recruitment by providing access to a pool of patients. Still, the patients recruited were only a smaller part of the list. These results are similar to those of other studies, which have shown that institutional resources improve patients’ access; however, they cannot fully address barriers related to eligibility and willingness to participate [42]. Consequently, institutional support is necessary but not sufficient, underscoring the importance of developing and maintaining an organizational network and communication with multiple institutional supports. Moreover, community-based recruitment yields better results, even with fewer participants overall. Although the volume was lower than that of the PRBB, community referrals highlight the trust and cultural relevance that partners bring to the process, as well as the importance of leadership engagement [43,44]. Similarly, evidence on Latino community engagement shows that trust is often stronger when recruitment comes from within the community [44]. The difference between institutional and community referrals points to both the promise and the limitations of relying on community partners at this early stage. Moreover, these findings suggest that a combination of both approaches is necessary to balance reach and depth in future CASA implementations.

The implementation process included the planning and champion construct, which relied on the training and recruitment outcomes. The training structure, the standardized recruitment procedures, and the CASA intervention protocol supported the planning construct. Pre- and post-training assessments served as fidelity tools, capturing changes in knowledge and reinforcing the importance of maintaining adherence to the CASA protocol. Additionally, participants’ feedback promotes continuous improvement of the training. On the other hand, when comparing referral sources, institutional recruitment yielded more participants. As such, the PRBB operated as an institutional champion for recruitment, enabling the examination of the benefits and limitations of relying on a single champion during the early stage. While institutional champions can enhance legitimacy and access, over-reliance may constrain recruitment diversity and scalability, underscoring the need to engage additional champions and stakeholders in future phases.

Taken together, the training results, recruitment patterns, and preliminary CASA outcomes illustrate how early implementation elements interact across CFIR domains and support the potential scalability of CASA intervention. Training supported individual readiness, recruitment outcomes reflected contextual constraints and resources, and preliminary patient and caregiver outcomes informed perceptions of intervention relevance. These interactions highlight the importance of early attention to implementation processes, particularly when delivering culturally adapted interventions in underserved populations.

These results prompt reflection on the ethical responsibilities and policy considerations involved in sustaining long-term implementation efforts. From an ethical and policy perspective, this study underscores the importance of fostering ongoing communication and education among community partners engaged in recruitment and training. Continued education for community partners may enhance adherence to ethical principles, such as informed consent and confidentiality, promote culturally responsive communication with patients, and reinforce the appropriate use of Motivational Interviewing techniques. Strengthening these ethical procedures aligns with best practices in implementation science and community-engaged research, particularly in settings serving underrepresented populations [45].

At a policy level, our findings highlight the need for coordinated institutional-community partnerships that balance regulatory compliance with culturally grounded engagement. The Puerto Rico Biobank’s role demonstrates how institutional infrastructures can support recruitment by facilitating access to eligible patients through data sharing, even when their role does not involve direct contact or recruitment activities. In contrast, community partners contributed to patient identification and initial engagement, emphasizing the complementary value of both approaches. Strengthening coordination between research teams and institutional sources, while continuing to empower community partners, may optimize recruitment efficiency and promote equitable access to research participation [46]. Future CASA implementation phases should include structured continuing education modules and periodic monitoring of recruitment processes to promote sustainability, ethical integrity, and long-term community engagement.

This study has some limitations, primarily related to its formative and exploratory nature. Notably, the small trainee samples limit the extent to which training feasibility findings can be generalized beyond this specific context. Additionally, measures used to evaluate knowledge related to both training programs were internally developed and aligned with training learning objectives, and did not undergo formal psychometric validation. As such, findings related to knowledge gains should be interpreted as indicators of training feasibility and content comprehension, rather than as validated measures of training effectiveness. Also, all trainee participants were female, which may limit the representativeness of their experiences and perspectives on training engagement and recruitment dynamics. Regarding intervention-related outcomes, we should interpret findings as preliminary therapeutic signals rather than definitive evidence of efficacy, despite statistically significant improvements. Longitudinal measures and larger samples are needed to determine long-term effects. However, these limitations reflect the early phase of implementation, where the primary goal was to assess feasibility and inform future iterations rather than achieve statistical generalizability. Moreover, recruitment relied mainly on a single institutional source—the Puerto Rico Biobank—which provided access to eligible patients but may not fully reflect other healthcare systems or community contexts. Future research should aim to reach broader and more diverse clinical populations by strengthening community partnerships and expanding Biobank recruitment pipelines. Scaling these strategies could support more equitable representation, increase reach, and enhance both the clinical applicability and sustainability of the CASA intervention.

## 5. Conclusions

These findings suggest that targeted training for doctoral students and community partners can support early implementation of the CASA intervention as reflected in the knowledge gains and participant feedback. The use of the CFIR framework provided a comprehensive structure for evaluating and strengthening pre-implementation training across intervention characteristics, individual factors, institutional settings, community engagement, and process during the early implementation. Although the small sample size limits generalizability, the findings offer important insight to refinement and scale-up training and recruitment strategies. Future work should focus on expanding stakeholder involvement, assessing the sustainability of knowledge gains over time, and further evaluating the CASA’s impact on patients and caregivers to promote more sustainable and equitable psychosocial support in cancer care, and exploring the possibility of including additional institutional sources.

## Figures and Tables

**Table 1:** Overview of the CASA Training.

Day	Content Focus	Activities	Duration
Group Training *			
1	Foundation of CASA: Introduction to MCP and CCST	Didactic lecture covering the individual adaptation for Latino/Hispanic populations of the Meaning-Centered Psychotherapy (MCP) and Communication Couple Skills Training (CCST). Guided role-plays were conducted for each MCP and CCST session included in CASA. Students were assigned to review the CASA manual in preparation for Day 2.	6–7 h
2	CASA intervention goals, sessions, and fidelity checklist	In-depth discussion of the CASA intervention manual. Review and clarification of clinical questions regarding the manual of the CASA intervention. Introduction and discussion of the fidelity checklist, emphasizing expectations for trainees. Role-plays focused on CASA Session 1, followed by supervisor feedback. Students continue to study the CASA intervention manual.	4 h
3	Role-plays on the CASA intervention.	Role-plays simulating CASA Sessions 2, 3, and 4, conducted under direct supervision with immediate feedback.	3 h
Individual Training			
4–5	Roleplays and individual supervision	One-o-one supervised roleplays focusing on CASA Session 5 and any other sessions where additional practice was required.	1–2 h

Note. * Each group training session included peer observation.

**Table 2:** Overview of the Recruitment Training.

Day	Content Focus	Activities	Duration
1	Ethical principles of human subject’s research and introduction to recruitment procedures	Overview of IRB requirements and confidentiality. Definitions of research, human subject, intervention, and interaction (45 CFR 46). Training on administration of the Distress Thermometer and interpretation. Introduction to Motivational Interviewing (MI) techniques: open-ended questions, reflective listening, affirmations.	2–3 h
2	Practice and application of recruitment and MI strategies	Supervised role-plays simulating recruitment scenarios (willing, ambivalent, and denying patients). Feedback and modeling by PI to reinforce empathy, validation, and autonomy support.	2–3 h

**Table 3:** Overview of the CASA intervention.

Session	Content Focus	Duration
1	Introductory Session to the meaning concept, sources of meaning and the history of cancer	45–60 min
2	Identity and communication skills as a messenger and a listener	45–60 min
3	Creative Sources of Meaning: Actively connecting with life	45–60 min
4	Experiential Sources of Meaning: Connecting with life through beauty, love and humor.	45–60 min
5	Transitions: Reflection and vision of future after the intervention	45–60 min

**Table 4:** Sociodemographic characteristics of the participants recruited and of the CASA intervention.

Variables	Participants Recruited (*n* = 52) *n* (%)	CASA Intervention	
		Patients (*n* = 11) *n* (%)	Caregivers (*n* = 11) *n* (%)
Age *	57	58	56
**Gender**			
Female	31 (59.6)	8 (72.7)	8 (72.7)
Male	21 (40.4)	3 (27.3)	3 (27.3)
**Marital Status**			
Married	27 (51.9)	5 (45.5)	7 (63.6)
Single	15 (28.8)	6 (54.5)	1 (9.1)
Live together	7 (13.5)	-	2 (18.1)
Divorced	2 (3.8)	-	1 (9.1)
Widowed	1 (1.9)	-	-
**Academic Level**			
High School	22 (42.3)	4 (36.4)	5 (45.5)
College	11 (21.2)	2 (18.2)	4 (36.4)
Master’s/Doctoral degree	5 (9.6)	1 (9.1)	2 (18.2)
Associate degree	9 (17.3)	3 (27.3)	-
Middle School	4 (7.7)	-	-
Six grade or less	1 (1.9)	1(9.1)	-
**Medical Insurance**			
Government	27 (51.9)	6 (54.5)	3 (27.3)
Private	17 (32.7)	2 (18.2)	6 (54.5)
Medicare	7 (13.5)	3 (27.3)	2 (18.2)
Do not have	1 (1.9)	-	-
Income *	$20,176	$23,367	$25,963
**Do you consider your income sufficient to cover your expenses?**			
No	31 (40.4)	4 (36.4)	6 (54.5)
Yes	21 (40.4)	7 (63.6)	5 (45.5)

Note. * We presented the means for age and income.

**Table 5:** CASA intervention outcomes.

Measures	Descriptive Statistics						Test Statistics			
	Pre-Assesesment			Post-Assesement			*Z*	Asymp. Sig (2-Tailed)	Adjusted α	Effect Size *r*
	Mean	SD	Mdn	Mean	SD	Mdn				
FACIT-SP-12	38.89	6.33	40.00	42.39	4.75	42.00	−2.618 ^a^	0.009	0.025	0.44
PHQ-8	6.94	4.49	7.50	3.28	3.16	2.00	−2.764 ^b^	0.006	0.0125	0.46
GAD-7	5.78	4.15	6.00	2.72	2.97	1.50	−2.667 ^b^	0.008	0.0166	0.44
DT	5.56	2.81	6.00	3.83	2.94	3.00	−2.195 ^b^	0.028	0.05	0.38
FACT G	72.94	17.20	70.50	82.29 *	13.67 *	81.00 *	−2.957 ^a^	0.003	0.01	0.51

Note. * *n* = 17; ^a^ = Based on negative ranks; ^b^ = Based on positive ranks.

**Table 6:** PR Bio-Bank recruitment frequencies and percentages.

Outcome	*n* = 96	%
Recruited	16	17.02
Do not meet inclusion criteria o were not interested	44	46.81
No answer	34	36.17

**Table 7:** Community partner referral frequencies and percentages.

Outcome	*n* = 11	%
Recruited	10	90.91
No answer	1	9.09

## Data Availability

The original contributions presented in this study are included in the article. Further inquiries can be directed to the corresponding author.

## Abbreviations

The following abbreviations are used in this manuscript:

CASA	Caregiver-Patients Support to Cope With Advanced Cancer
MCP	Meaning Centered Psychotherapy
CCST	Communication Couple Skill Training
CFIR	Consolidated Framework for Implementation Research
PRBB	Puerto Rico Bio-Bank
MI	Motivational Interviewing
FACIT-SP-12	Functional Assessment of Chronic Illness Therapy-Spiritual Well-being 12
PHQ-8	Patient Health Questionnaire-8
GAD-7	Generalized Anxiety Disorder-7
DT	Distress Thermometer
FACT-G	Functional Assessment of Cancer Therapy
